# Epidemiology of Dengue in Argentina during the 2010/11 to 2019/20 Seasons: A Contribution to the Burden of Disease

**DOI:** 10.3390/tropicalmed9020045

**Published:** 2024-02-10

**Authors:** Solana Rapaport, Mariana Mauriño, María Alejandra Morales, Cintia Fabbri, Victoria Luppo, María Pía Buyayisqui, Teresa Varela, Carlos Giovacchini, Analía Urueña

**Affiliations:** 1 Centro de Estudios para la Prevención y Control de Enfermedades Transmisibles, Universidad Isalud, Venezuela 931, Ciudad Autónoma de Buenos Aires C1095AAS, Argentina; 2 Dirección de Epidemiología, Ministerio de Salud de la Nación, Av. 9 de Julio 1925, Ciudad Autónoma de Buenos Aires C1073ABA, Argentina; 3 División Arbovirus, Departamento Diagnóstico Laboratorial y Referencial, Instituto Nacional de Enfermedades Virales Humanas “Dr. Julio I. Maiztegui” (INEVH)-ANLIS, Monteagudo 2510, Pergamino 2700, Argentina

**Keywords:** dengue, epidemiological trends, Argentina

## Abstract

Background: Dengue is an important public health problem in Argentina, as in many other countries. We reviewed and updated information on the dengue disease burden in Argentina over a 10-year period. Methods: We conducted a retrospective descriptive study from 2010 to 2020 based on data from the National Health Surveillance System. The main outcomes included dengue cases, incidence rates, deaths, and serotype distribution by season, age group, and region. Results: A total of 109,998 confirmed cases of dengue were reported. Seasonality stands out, prevailing during summer and autumn. Two main outbreaks (seasons 2015/16 and 2019/20), with increasing magnitude, were observed. The 2019/20 season showed the highest number of cases (58,731) and incidence rate (135/100,000). The Northeast region had the highest number of cases and incidence rate. In 2020, for the first time, autochthonous cases were registered in the Cuyo region. The only region with no autochthonous cases was the South. Adolescents and young adults showed the highest incidence rate. The case fatality rate for the period was 0.05%. Four serotypes circulated, but the predominant one was DEN-1 (78%). Conclusions: Dengue has been expanding temporally and spatially. Although the DEN-1 serotype widely predominated, the increasing circulation of other serotypes raises concerns regarding re-exposure and the severity of future cases. Understanding epidemiological trends is key to defining public prevention and control policies.

## 1. Introduction

Dengue is a viral infection transmitted by mosquitoes and is currently the most important arbovirus worldwide in terms of morbidity and mortality. Dengue virus belongs to the Flaviviridae family, and there are four variants known as serotypes 1, 2, 3, and 4. Immunity is serotype-specific, meaning that infection with a specific serotype provides permanent immunity against that serotype (homologous immunity), but only temporary immunity against the other serotypes (heterologous immunity) is observed [1]. Subsequent infections caused by different serotypes increase the risk of severe dengue [2].

In the Americas, the main vector responsible for dengue transmission is the *Aedes aegypti* mosquito. The simultaneous presence of the vector, infected and viremic individuals, and susceptible hosts is needed for transmission. Transmission occurs when a vector mosquito bites a viremic person, incubates the virus for a period (between 8 and 12 days), and then bites a susceptible person, transmitting the infection to them. Transmission from a pregnant person to their baby has also been documented, but the risk seems to be low [2].

Currently, the disease is endemic in more than 100 countries in the regions of Africa, the Americas, the Eastern Mediterranean, Southeast Asia, and the Western Pacific. It is estimated that there are between 100 and 400 million infections each year, with an increasing incidence in recent decades. Moreover, according to estimates by the World Health Organization (WHO), about half of the world’s population is at risk of contracting dengue [2].

There are various factors associated with this increase, including climate change, rapid and unplanned population growth in urban areas, inadequate provision of safe water and solid waste management, as well as the risk from travel and migration to endemic areas and inadequate vector control [1,3,4].

In the Americas region, the number of dengue cases has also increased in the past 4 decades, from 1.5 million cumulative cases in the 1980s to 16.2 million in the 2010–2019 decade. All four serotypes of dengue circulate throughout the region, and in some cases, they circulate simultaneously, which increases the risk of severe dengue [5].

In 2019, the highest number of dengue cases worldwide was recorded. All regions of the world were affected. It was also the year with the highest number of reported cases in the history of dengue in the Americas, with more than 3.1 million cases, including 28,203 severe cases and 1773 deaths [6]. Of the total reported cases, 0.9% were classified as severe dengue, and the case fatality rate was 0.049% [7]. However, it is possible that the 2023 season will reach or even exceed that of 2019, since up to June there were more than two million dengue cases notified. In this season, the South Cone subregion reached the highest cumulative incidence of 564 cases/100,000 inhabitants [6]. 

In Argentina, the *Aedes aegypti* mosquito was eradicated in 1963 thanks to successful vector control programs. However, its presence was detected again in 1987 in the northern part of the country, and years later, in some provinces in the central region [8]. During the last decade, it has also been detected in locations below the known transmission limit of the virus, but this occasional detection did not necessarily lead to the establishment of a mosquito population. A clear example is the finding of *Aedes aegypti* through ovitraps in the city of Neuquén (South Region) in 2010, followed by subsequent negative monitoring in the following years [9]. *Aedes albopictus* was also detected in some departments in Argentina [10], and it causes concern, considering this species has been recognized as the second vector for transmitting dengue. 

On the other hand, in 1997, the first autochthonous transmission of dengue was reported since the mosquito eradication, indicating the reemergence of dengue in the country [11]. Since then, successive outbreaks have occurred during the warmer months, closely related to the epidemiological situation in neighboring countries [12,13], and by 2009, the first major dengue epidemic occurred, caused by serotype DEN-1 and affecting 15 jurisdictions, including for the first time the central and most populated region, with over 26,000 autochthonous cases [14]. 

In Argentina, dengue is a mandatory notifiable event throughout the territory for all healthcare providers. Dengue surveillance is carried out under an integrated modality, which includes clinical, epidemiological, and laboratory data. Since 1997, the National Institute for Viral Human Diseases (INEVH-ANLIS) has been the National Reference Center for the diagnosis of dengue and other arboviral diseases in Argentina [15], which works together with a national laboratory network in the affected provinces.

Like in other countries in the region and around the world, dengue is an important public health problem in Argentina, and studies quantifying the burden of the disease are limited. The general objective of this study is to contribute to the understanding of the burden of dengue in Argentina, providing an overview of dengue during a ten-year period, which can be useful for decision makers in public health policies.

## 2. Materials and Methods

A descriptive study based on secondary data was conducted to describe the epidemiology and burden of dengue in Argentina over a ten-year period. Each dengue season spans from epidemiological week (EW) 31 of a given year to EW 30 of the following year. The analyzed period covered EW 31 of 2010 to EW 30 of 2020.

### 2.1. Data Sources and Analysis

Dengue cases, deaths, and serotypes were provided by the Directorate of Epidemiology of the Ministry of Health, which consolidates information from the National Health Surveillance System (SNVS). Immediate notification to the SNVS is required for suspected cases, which will be considered confirmed if they have a laboratory-confirmed dengue diagnosis, or, during an outbreak, if they have compatible clinical symptoms and an epidemiological link to laboratory-confirmed cases. The laboratory diagnostic method used, as NS1 antigen, IgM antibody detection, molecular detection of the viral genome, or virus isolation and PRNT seroconversion, varied depending on the time of sample collection, the availability of these methods at the local laboratory, the epidemiological situation, and the current diagnostic algorithm at the moment of the event [15,16,17,18]. Finally, for clinical and epidemiological surveillance purposes, dengue is classified as with or without warning signs, and severe dengue, following the classification proposed by the WHO in 2009 [19].

Confirmed dengue cases reported to the SNVS from EW 31 of 2010 to EW 30 of 2020 were considered for this analysis. Since 2018, the surveillance system has incorporated integrated clinical, epidemiological, and laboratory information, whereas in the past, cases were reported using two separate systems: one for clinical–epidemiological data and another for laboratory data. Thus, the system needed integration and preliminary analysis before case-counting. For this study, dengue cases up to the year 2018 were obtained in a grouped manner, matched up by EW, department, and age groups. Since the year 2019, information was reported individually.

For these cases, age, legal gender, jurisdiction of residence, history of travel, EW, and final case classification (dengue with or without warning signs, or severe dengue for cases reported to the SNVS) were analyzed. 

Dengue hospitalizations for the years 2010 to 2018 were provided by the Directorate of Statistics and Health Information (DEIS) of the Ministry of Health, which collects information from discharge records completed and coded by the 24 jurisdictions of the country according to the International Classification of Diseases (ICD-10) [20]. At the moment the study was conducted, this information was available up to the year 2018, so hospitalized cases for the period 2019–2020 were obtained from the variable “hospitalization” of confirmed dengue cases notified using the surveillance system (SNVS).

Circulating serotypes were analyzed for those cases with serotype identification reported to the SNVS between EW 31 of 2010 and EW 30 of 2020.

### 2.2. Outcomes 

Dengue cases, hospitalizations, deaths, incidence, and mortality rates were stratified by season and age groups: 0 to 4 years old, 5 to 14 years old, 15 to 24 years old, 25 to 44 years old, 45 to 64 years old, and 65 or older. Outcomes were also disaggregated by regions: Argentine Northeast (NEA), Argentine Northwest (NOA), Center, Cuyo, and South region. 

Dengue-related incidence and mortality rates by season were expressed by 100,000 inhabitants and were estimated considering confirmed dengue cases and deaths, respectively, and the population at risk during that season. Cumulative dengue incidence by age group and region was also expressed by 100,000 inhabitants and estimated considering the confirmed dengue cases among the total period and the population at risk for that age group and region. Population estimates based on the 2010 Census published by the National Institute of Statistics and Census were used to calculate incidence and mortality rates.

The analysis estimated dengue serotype proportions for the whole period by season and region. 

### 2.3. Ethics Considerations

In all cases, the obtained information was anonymized, and confidentiality was maintained in accordance with all applicable subject privacy requirements and the guiding principles of the Helsinki Declaration.

The protocol was submitted for evaluation and received approval from the Clinical Research Ethics Committee (CEIC)—Stamboulian (Registration Code 7540) and is registered in the PLIISA Platform of the Government of Buenos Aires City.

## 3. Results

### 3.1. Dengue Cases and Incidence 

**Figure 1:**
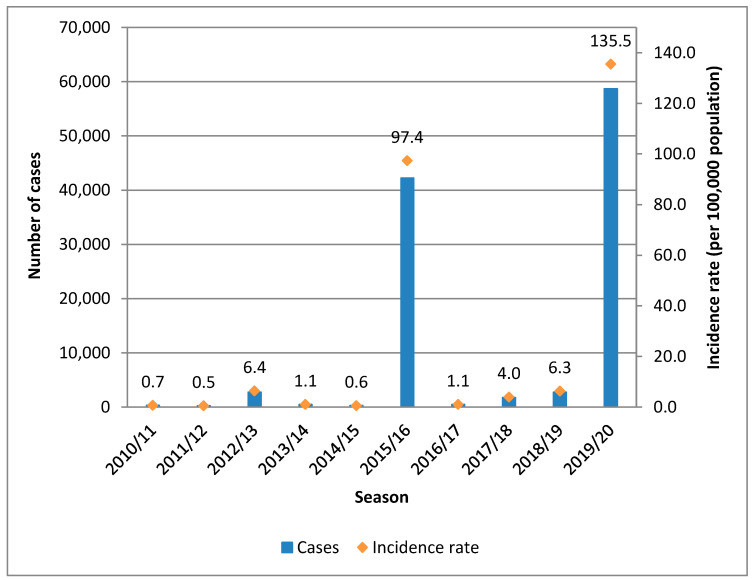
Dengue cases and incidence rate by season. Argentina. Seasons 2010/11 to 2019/20. *N* = 109,998. Source: authors’ own elaboration based on data from the SNVS and SNVS 2.0, National Ministry of Health.

**Figure 2:**
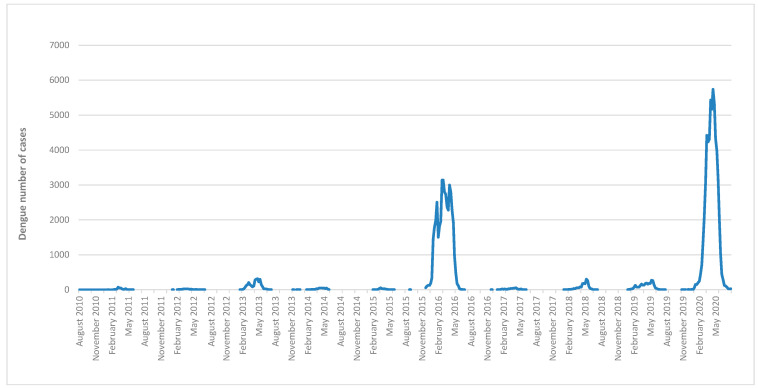
Cases by epidemiological week. Argentina. Seasons 2010/11 to 2019/20. Source: authors’ own elaboration based on data from the SNVS and SNVS 2.0, National Ministry of Health.

A total of 109,998 cases of dengue were reported to the SNVS between EW 31/2010 and EW 30/2020 (seasons 2010/11 to 2019/20). During the 2019/20 season, there were 58,731 recorded cases, accounting for 53% of the cases in the analyzed decade and the highest incidence rate observed during that period (135 cases per 100,000 inhabitants). A total of 99.9% of the cases in this season occurred between epidemiological weeks 1 and 30 of 2020. The 2015/16 season followed in magnitude, with 42,215 cases (38% of the cases in the decade) and an incidence rate of 97 cases per 100,000 inhabitants (Figure 1). The seasons with the highest number of cases in Argentina (2019/20 and 2015/16) coincide with the main outbreaks that occurred in the Americas, particularly in the Southern Cone subregion. (Supplemental Figure S1). The country is in the southern hemisphere and extends from latitude 21° to 55° from North to South. The climate is divided into four well-defined seasons: summer (December–March), autumn (March–June), winter (June–September), and spring (September–December). Dengue seasonality stands out, prevailing in the cases during the summer and autumn, with transmission interruption during winter (Figure 2).

Cases of severe dengue could only be analyzed for the first half of 2019 and the 2019/2020 season, as cases from previous seasons were obtained in a grouped form. During those 18 months, the proportion of severe dengue cases recorded was 0.06%, and cases with warning signs comprised 0.79%.

**Figure 3:**
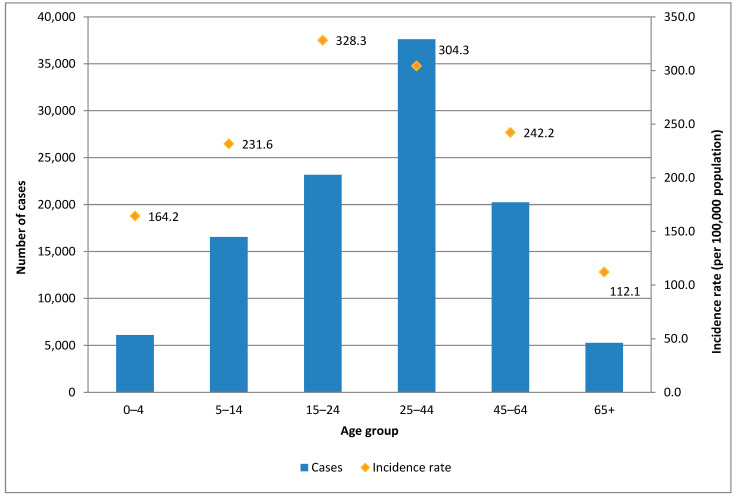
Dengue cases and cumulative incidence rate by age group. Argentina. Seasons 2010/11 to 2019/20. *N* = 109,998. Source: authors’ own elaboration based on data from the SNVS and SNVS 2.0, National Ministry of Health.

The age group between 25 and 44 years old showed the highest number of cases in the analyzed period (34.2% of the total). The group of 15- to 24-year-olds followed it, representing 21% of the total for the period. A similar proportion of cases was observed in the 45- to 64-year-old group (18.4%); the 5- to 14-year-old group accounted for just over 14%, and finally, the extremes of life (<5 years old and ≥65 years old) represented around 5% of the total cases each. Since these age groups are not homogeneous in terms of the number of cohorts and individuals included in each group, a cumulative incidence rate for the entire period was estimated to better represent the impact of dengue in each age group, considering the total number of cases for the entire period and the average population in each age group. Thus, the age group of teenagers and young adults (15 to 24 years old) had the highest cumulative incidence rate for the total period, reaching 328.3 cases per 100,000 inhabitants. They were followed by working-age adults and then children aged 5 to 14 years old (Figure 3). This pattern was consistently observed across almost all seasons, irrespective of the case count. A subtle variation in the age distribution of cases was noted during the 2010/11 and 2015/16 seasons, wherein a secondary peak in cases among the 0–4 age group occurred that followed in magnitude the peak observed in adolescents and young adults.

In the analyzed period, the NEA region had the highest number of cases and, at the same time, the highest cumulative incidence rate (45,767 cases and 1143 cases per 100,000 inhabitants, respectively). The Center region followed in the number of cases, although because of the larger population, the cumulative incidence rate was lower than the NOA region (35,344 cases and 125.7 cases per 100,000 inhabitants, respectively). The NOA region, despite having a lower number of total cases, had a higher cumulative incidence rate (27,792 cases and 554.9 cases per 100,000 inhabitants, respectively). Finally, the Cuyo region showed the lowest number of cases and cumulative incidence rate (1059 cases and 30.4 cases per 100,000 inhabitants, respectively).

**Figure 4:**
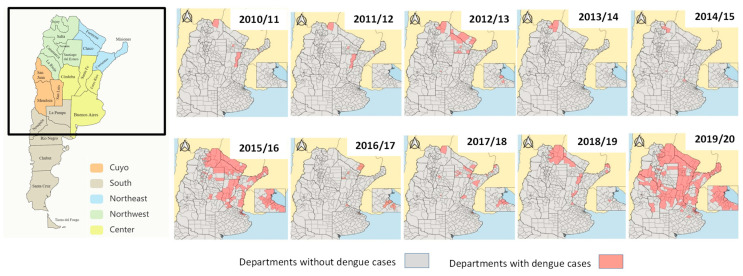
Argentine regions. The map on the left describes Argentinian regions and jurisdictions. The black square represents the selected area shown in the maps by season on the right. Maps by season show The departments with autochthonous dengue cases. Argentina. Seasons 2010/11 to 2019/20. Source: authors’ own elaboration based on data from the SNVS and SNVS 2.0, National Ministry of Health.

The 2019/20 season, in addition to having the highest number of cases in the studied period, had the broadest geographical impact, involving NOA, NEA, Center, and Cuyo regions for the first time with autochthonous transmission of dengue (Figure 4). The South region only reported cases from travelers during this period, which were excluded from this analysis. 

This territorial expansion can be clearly observed when the number and location of departments and provinces with autochthonous dengue cases are analyzed throughout the entire period. Thus, out of the two main outbreaks, provinces with dengue ranged between 5 and 12. However, during the 2015/16 and 2019/20 seasons, the disease expanded from North to South and West, and autochthonous dengue cases were reported in 15 and 16 provinces, respectively (Figure 4).

### 3.2. Dengue-Related Hospitalizations

There were 4749 hospitalizations due to dengue in the analyzed period. Of the total, 3161 hospitalizations were reported by the DEIS until the year 2018. A total of 98% of the discharge diagnoses corresponded to the ICD-10 code A90 (classic dengue) and 2% to A91 (hemorrhagic dengue). Of the other 1588 hospitalizations reported in 2019 and 2020 (up to EW 30) by the SNVS, 92.3% of the events corresponded to cases of dengue without warning signs, 6.7% to dengue with warning signs, and 1% to severe dengue.

Almost half of the dengue-related hospitalizations were reported in the 2015/16 season (45.0%), followed by the 2019/20 season, with 30.6%. The highest number of dengue-related hospitalizations was observed in the 25 to 44 age group (1428 cases, 30%). The age groups at the extremes of life had the fewest hospitalization cases, with 303 in the 0 to 4-year-old group and 345 in the 65+-year-old group. Once again, to better represent the impact by age group, a hospitalization rate per 100,000 population was estimated for each age group, being the highest for the 15 to 24 age group (12.8 per 100,000 population) and the lowest for the 65+ group (7.3 per 100,000 population) (Supplemental Figure S2).

Although 50.3% of the hospitalizations were recorded in the Central and populated region (2390 cases), the cumulative hospitalization rate for the entire period considering the population exposed followed the same pattern as the cumulative incidence rate and was the highest in the NEA region (27.5 versus 23.8, 8.5, and 1.4 per 100,000 population in the NOA, Center, and Cuyo regions, respectively). On the other hand, if considering hospitalized cases over the total dengue cases in each region, the Center region showed a ratio of 0.067, which was higher than in the NOA region (0.043) and in the NEA region (0.024). However, since the dengue cases and hospitalization data were sourced from different registries, these ratios should be interpreted with this limitation, considering that both samples may not correlate in their entirety. 

### 3.3. Dengue-Related Deaths and Case Fatality Ratio (CFR)

There were 43 deaths due to dengue reported to the SNVS in the analyzed period. The 2019/20 season showed the highest number of death cases (26) and the highest mortality rate (0.06 per 100,000 inhabitants), followed by the 2015/16 season (13 deaths and mortality rate of 0.03 per 100,000 inhabitants). Finally, the overall CFR for the period was 0.05%, 0.07% in the Center region, 0.01% in the NEA region, and 0.05% in the NOA region. The CFR also increased with age, reaching the highest value (0.21%) in the 65 years or older age group (Supplemental Figure S3).

### 3.4. Circulating Serotypes

Serotype identification was available for 9567 samples between 2010/11 and 2019/20. A total of 78.5% of these determinations (7508) corresponded to serotype DEN-1, 16.2% (1549) to serotype DEN-4, 5.1% (487) to serotype 2, and 0.2% (23) to serotype DEN-3. 

Serotype DEN-1 was the only one detected in all analyzed seasons and predominated in almost all of them, except in 2011/12, 2012/13, and 2014/15. In 2011/12 and 2012/13, serotype DEN-2 was the most frequently identified, and in 2014/15, it was DEN-4. The 2016/17 season was the only one in which a single serotype (DEN-1) was detected. In the others, between two and four different serotypes were isolated, although in different proportions (Supplemental Figure S4).

**Figure 5:**
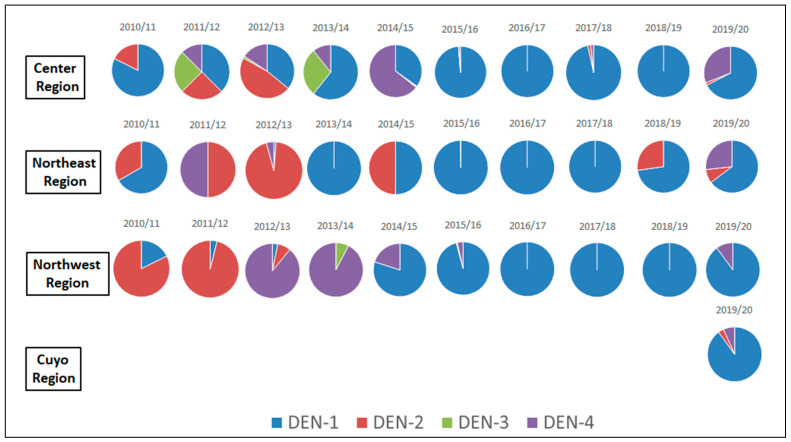
Proportional distribution of identified dengue virus serotypes by region. Argentina. Seasons 2010/11 to 2019/20. *N* = 9567. Source: authors’ own elaboration based on data from the SNVS. National Ministry of Health.

Serotype DEN-1 predominated in all age groups and in all regions of the country. However, the pattern of serotype circulation varied by region, and in the same season, a different serotype could have predominated in one or another region (Figure 5). Furthermore, during some seasons, certain serotypes were only identified in a specific region. For example, in the 2011/12 season, DEN-3 was only identified in the Central region, while in the 2018/19 season, DEN-2 was only identified in the NEA region.

## 4. Discussion

Since the re-emergence of dengue in Argentina in 1997, outbreaks have been recorded almost every year, except in 2001 and 2005, with a heterogeneous spatial and temporal distribution in the affected territory [21]. The objective of this analysis was to review the disease burden of dengue over a 10-year period from the 2010/11 to 2019/20 seasons by gathering information from official sources. During this period, nearly 110,000 confirmed cases of dengue were reported to the National Health Surveillance System throughout the country, and cases were notified with a defined seasonal pattern during the summer and autumn and a stop during winter. In this cycle, two epidemic seasons stood out: 2015/16 and 2019/20. The latter confirmed 53% of the cases over the 10-year period, with autochthonous cases reported in 4 out of the 5 regions of the country, most of them in the Central region, representing the largest epidemic outbreak in the history of our country up to that moment. This trend observed in Argentina, with increasingly larger outbreaks, is similar to that reported by other countries and in the Americas globally, highlighting the significant public health problem that dengue represents in our continent and in the world [4,22].

The year 2019 was the one with the most cases in the American Region. However, in our country, 99.9% of the cases in the 2019/2020 season occurred in the first semester of 2020 during the summer and autumn. This later outbreak suggests that in Argentina, migratory movements from neighboring countries continue to play a key role in the onset of the outbreaks, beginning with imported cases that are later transmitted and disseminated locally, given the presence of the mosquito and the favorable climatic and demographic conditions. In this regard, Carbajo et al. analyzed the environmental conditions and the spatial–temporal pattern of dengue cases and observed that the number of dengue cases during the warm months of one season was associated with the temperature conditions of the previous autumn and the viral circulation in neighboring endemic countries [23].

To highlight, in March 2020, coinciding with the peak of the dengue outbreak, the first cases of COVID-19 were detected in Argentina [24], and it was the beginning of a new challenge. The healthcare system had to direct resources and efforts to respond to the pandemic, and although surveillance for both diseases was recommended and maintained [25], the diagnosis of SARS-CoV-2 may have been prioritized in the management of febrile patients. Although by the end of the dengue outbreak not all cases required a laboratory diagnosis to be considered a confirmed case of dengue, over-reporting was unlikely. The absence of respiratory symptoms as clinical criteria for a suspected dengue case and the requirement for a laboratory-confirmed epidemiological link to dengue makes it improbable that many cases of COVID-19 could have been erroneously considered confirmed dengue cases. On the contrary, the overlap of symptoms, lack of knowledge about this new disease, and people’s fear of getting infected with COVID-19 may have resulted in a delay and/or decrease in dengue outpatient visits. Consequently, it is possible that the total number of dengue cases in that year was higher than those registered by the epidemiological surveillance system, as well as the coexistence of both infections in the same patient may have occurred [26,27]. In this regard, as alerted by the Pan American Health Organization, the role of epidemiological surveillance is crucial, as well as having clinical management protocols for acute febrile diseases based on a scenario of co-circulation of arboviral diseases with other febrile diseases such as measles, COVID-19, and other respiratory illnesses (e.g., influenza) [28].

Regarding dengue disease in the different age groups assessed in this analysis, adolescents and young adults had the highest cumulative incidence rates over a 10-year period and during seasons with higher reported cases, similar to what occurs in other countries in the region [29,30,31]. The age groups at the extremes of life (children aged 0 to 4 years old and individuals aged 65 or older) reported the lowest number of dengue cases. However, the impact in older adults was the greatest, with the highest dengue cumulative mortality and CFR throughout the entire period. The higher CFR in the elderly is likely related to underlying conditions, as demonstrated in the 2002 outbreak in Kaohsiung, Taiwan [32]. In this outbreak, the highest dengue fatality occurred in individuals over 55 years old with underlying conditions such as high blood pressure, chronic kidney failure, or diabetes. It is important to continue close epidemiological surveillance and analyze whether this age distribution remains consistent in a country where endemicity has not yet been determined or if there are variations in the trend, as reported in different regions and seasons in Brazil [29].

Regarding the geographical distribution of cases, the NEA region had the highest number of cases and cumulative incidence rate throughout the entire analyzed period. It was also the most affected during the 2015/16 season. However, in the last and largest outbreak, during the 2019/20 season, the Central region recorded the highest number of cases, and the NOA region showed the highest incidence rate. The territorial expansion of dengue began to be evident following the first major outbreak in 2009. Previously, dengue transmission was limited to provinces in the northern part of the country with tropical and subtropical climates. Subsequently, there was an increasing trend of dengue cases in central jurisdictions with temperate climates, such as the Autonomous City of Buenos Aires, Buenos Aires Province, Córdoba, and Santa Fe [9,11,33]. Already in the 2019/20 season, autochthonous cases were recorded for the first time in the Cuyo region, particularly in San Luis province, where no previous outbreaks had been reported.

A correlation between the incidence of dengue fever and climate conditions or mosquito vector density was out of the scope of our study. However, some authors have analyzed and compared the variations in temperature and precipitations with vector density or the incidence of dengue in our country. As an example, López et al. reported significant differences in the mean, maximum, and minimum annual temperatures between periods with and without dengue transmission, but no significant differences in the precipitations of both periods were observed [34]. They also describe an increase in the number of days with optimal temperatures for DENV transmission in 60% of the provincial capitals, especially those located in the Central and Cuyo regions, where dengue cases have increased in the last decade. Another longitudinal vector surveillance study conducted by Estallo and collaborators in the temperate city of Córdoba (central region of Argentina) found an increase in the proportion of homes with juvenile *Aedes aegypti* over the study period (from 5.7% of homes in 2009/10 to 15.4% of homes in 2016/17) [35]. In addition, they reported a positive correlation between eggs per ovitrap and larval abundance with mean temperature in the same month. However, in this study, autochthonous dengue was not positively associated with vector or climate variables, and the authors suggest that other factors, such as movements of viremic humans, may have played a more important role in dengue transmission during this period. Nevertheless, given the higher population density and the vector’s increasing presence and adaptation to temperate climates [36,37], it is expected that this southern expansion trend of dengue will continue to extend, and in future outbreaks, the Central region will likely contribute increasingly to the number of cases.

Hospitalizations due to dengue were observed in all seasons, following a similar pattern to that of cases in terms of territorial distribution and age groups. The higher ratio of hospitalization over the total number of cases in the Center region, compared to the NEA and NOA, could be attributed, in part, to the broader availability and accessibility of secondary and tertiary healthcare facilities. Simultaneously, it could be linked to the comparatively limited experience of health teams in the management of dengue cases, potentially resulting in increased hospitalization as a preventive measure. It is noteworthy that in the 2019/2020 season, fewer hospitalizations were reported compared to the previous outbreak in 2015/16. This situation could be attributed, on the one hand, to the limitations of using different sources of information for this outcome and the possible under-reporting of this variable in the recently implemented surveillance system. On the other hand, it is possible that in 2020, along with the COVID-19 pandemic, the focus on hospitalization for respiratory conditions may have led to fewer dengue patients being admitted. Nevertheless, the number of hospitalizations and deaths due to dengue during this period was generally low, highlighting that, in our country up to 2020, this disease was characterized mostly by its morbidity, costs, and burden on the healthcare system during each season, especially during outbreaks. Additionally, deaths due to dengue during this period occurred proportionally more often in older adults, likely linked to coexisting conditions as described earlier, with a low fatality rate for the whole population (<0.05%), similar to that reported by the Pan American Health Organization (PAHO) for the region [38].

Regarding dengue serotype circulation, there was a marked predominance of DEN-1 in Argentina during the analyzed period as in the rest of the Latin American region [38]. However, co-circulation of two, three, and even all four serotypes was observed in some seasons in the same Argentinian region, even though not always in the same locality or department. The circulation of different serotypes over time poses a higher risk of severe dengue and fatality in the future, as suggested by some authors [39,40], and it was considered one of the five criteria for identifying populations at risk of severe dengue by departments in Argentina in a study by Varela et al. [21].

This study has some limitations. The National Health Surveillance System (SNVS) relies on clinical information provided by healthcare professionals who assist and notify symptomatic cases, as well as microbiological information provided by the growing network of laboratories that diagnose arboviruses in all jurisdictions of the country. In this sense, the unification of clinical and laboratory data in the surveillance system (SNVS 2.0) since 2018 is a fundamental pillar for strengthening the surveillance of all communicable diseases and improving the quality of reports. However, it is likely that the disease burden of dengue is higher than reported in this analysis. Like other passive surveillance systems, asymptomatic or mild cases who do not seek medical care are not captured by the healthcare system. Additionally, there may be cases in which healthcare teams do not suspect dengue in the presence of nonspecific febrile illness, as there could also be suspected/confirmed cases that are not properly reported. The first situation could occur especially outside epidemic periods when community awareness and perception of risk decrease, and the second one during outbreak seasons due to healthcare system overload and prioritization of patient assistance over-reporting or in areas where the community deals with dengue every season, normalize the situation, and do not seek medical care for each episode.

Hospitalizations may also be underestimated. The consolidated data until 2018 only include public healthcare facilities, so hospitalizations in private and social security subsectors are not reflected. Furthermore, under-coverage was reported in some jurisdictional archives during the analyzed period. In addition, hospitalizations in 2019 and 2020 were obtained from the SNVS, with the likely underreporting mentioned earlier. Understanding that these limitations are inherent to many passive information systems, our data are reliable and comparable to those of other countries in the region and the world.

In our analysis, the identification of severe dengue cases through the SNVS for the 2019/2020 season was very limited. This could be due to a deficit in recording this variable in the “signs and symptoms” section of the surveillance system, which was incorporated in 2018 and was still being implemented during the period included in this study. Nevertheless, the proportion of severe dengue in the Americas has been decreasing in the last decade, going from 3% to <1% in the last 5 years [38]. Therefore, considering this trend and the low number of hospitalizations recorded in our information systems, it is likely that the proportion of severe dengue cases in Argentina is in line with the reported proportion for the region.

Finally, there was no serotype information available for all hospitalized or fatal cases, so we could not identify associations between serotypes and other variables. This study was conceived as an observational investigation with the primary goals of consolidating data from various official sources over a substantial period and describing dengue trends in Argentina. While the detailed characterization of variables associated with key findings and the formulation of hypotheses explaining diverse behaviors—especially regarding the severity and lethality of dengue across departments, age groups, and circulating serotypes—were beyond the scope of this study, these insights will undoubtedly prove valuable for guiding future research.

In summary, this analysis provides an overview of ten years of dengue epidemiological surveillance in Argentina and highlights the complexities of a disease that is expanding temporally and spatially throughout our national territory, along with the growing exposed population. While the circulation of the DEN-1 serotype has been predominant during this period, the increasing circulation of other serotypes raises concerns regarding the potential re-exposure and severity of future cases.

This review also highlights the strengths and weaknesses of the surveillance and registration systems for cases, hospitalizations, and deaths. In this regard, Argentina has a unified epidemiological surveillance system for the entire country—the SNVS—which provides real-time information from different healthcare facilities (public, private, and social security) and, since 2018, has integrated clinical and laboratory components into a single information system, directing information to the stakeholders involved in surveillance and control. In addition, a consolidated network of laboratories is distributed throughout the territory in areas at risk of dengue transmission, with local serological and molecular diagnostic capabilities, as well as a differential diagnosis for other flaviviruses and arboviruses, which provides high sensitivity and specificity to the surveillance system. In the context of a complex disease and the increasing number of arboviruses circulating in the Americas region, it is of high relevance for the future to sustain these achievements, to keep training and support teams to improve data quality collection, as well as to strengthen steadily the national laboratory network.

Finally, we remark on the importance of understanding the patterns of disease burden distribution and serotype circulation of dengue, as well as their trends over time, as a fundamental tool for identifying populations at highest risk and consequently implementing targeted measures of environmental sanitation, vector control, and community education. Moreover, this information aims to serve as a key input for decision makers to direct future prevention actions, including vaccination.

## Data Availability

Data was obtained from Argentinian official sources and are available in https://datos.gob.ar (accessed on 1 September 2023) and upon request in https://tramitesadistancia.gob.ar/tramitesadistancia/inicio-publico (accessed on 1 September 2023).

## Supplementary Materials

The following supporting information can be downloaded at: https://www.mdpi.com/article/10.3390/tropicalmed9020045/s1, Figure S1: Number of dengue cases reported in the South Cone Region (A) and in Argentina (B); Figure S2: Dengue-related hospitalizations and cumulative hospitalization rate by age group. Argentina. Seasons 2010/11 to 2019/20; Figure S3: Case fatality rate by age group. Argentina. Seasons 2010/11 to 2019/20; Figure S4: Identified dengue virus serotypes by season. Argentina. Seasons 2010/11 to 2019/20. N = 9567.
