# Supplementary material for: Epidemiology of Dengue in Argentina during the 2010/11 to 2019/20 Seasons: A Contribution to the Burden of Disease
Source: Trop Med Infect Dis. 2024 Feb 10;9(2):45. doi: 10.3390/tropicalmed9020045 (PMC10891897; doi:10.3390/tropicalmed9020045)
Supplement: Supplementary file 1 [file tropicalmed-09-00045-s001.zip › tropicalmed-2642712-supplementary.pdf]

Supplemental material

Figure S1. Number of dengue cases reported in the South Cone Region (A) and in Argentina (B).

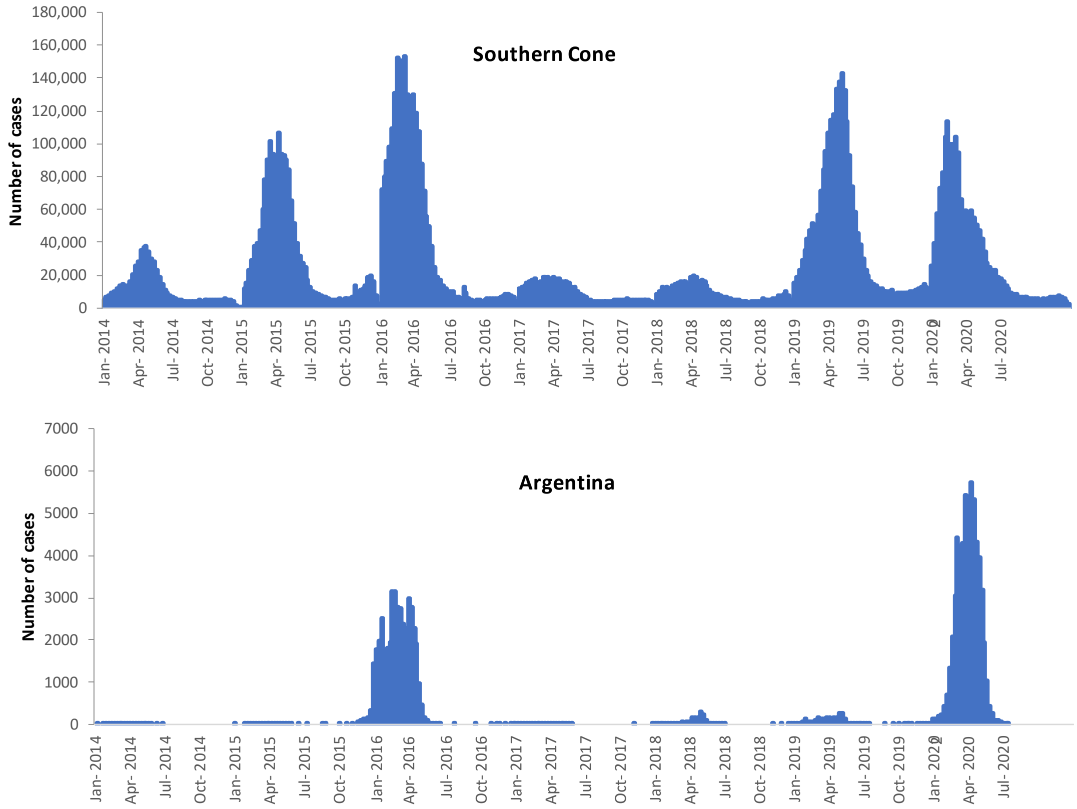

Source: Authors' own elaboration based on data from PAHO's Health Information Platform for the Americas (PLISA) and the SNVS and SNVS 2.0. National Ministry of Health.

Figure S2. Dengue-related hospitalizations and cumulative hospitalization rate by age group. Argentina. Seasons 2010/11 to 2019/20.

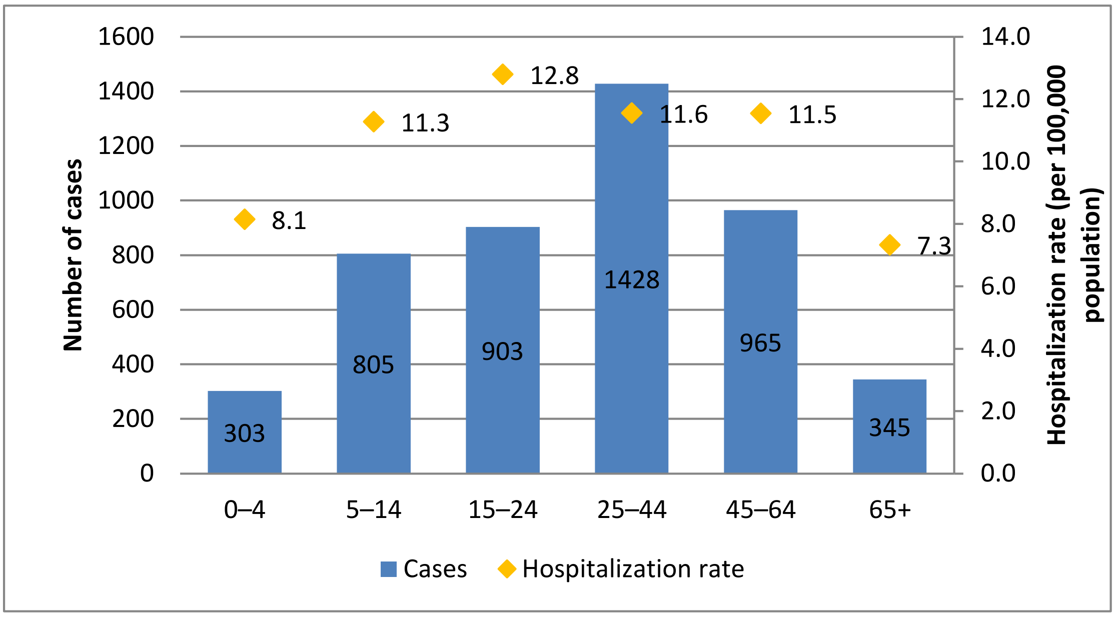

Source: Authors' own elaboration based on data from the SNVS and SNVS 2.0. National Ministry of Health.

Figure S3. Case fatality rate by age group. Argentina. Seasons 2010/11 to 2019/20. *N*=43

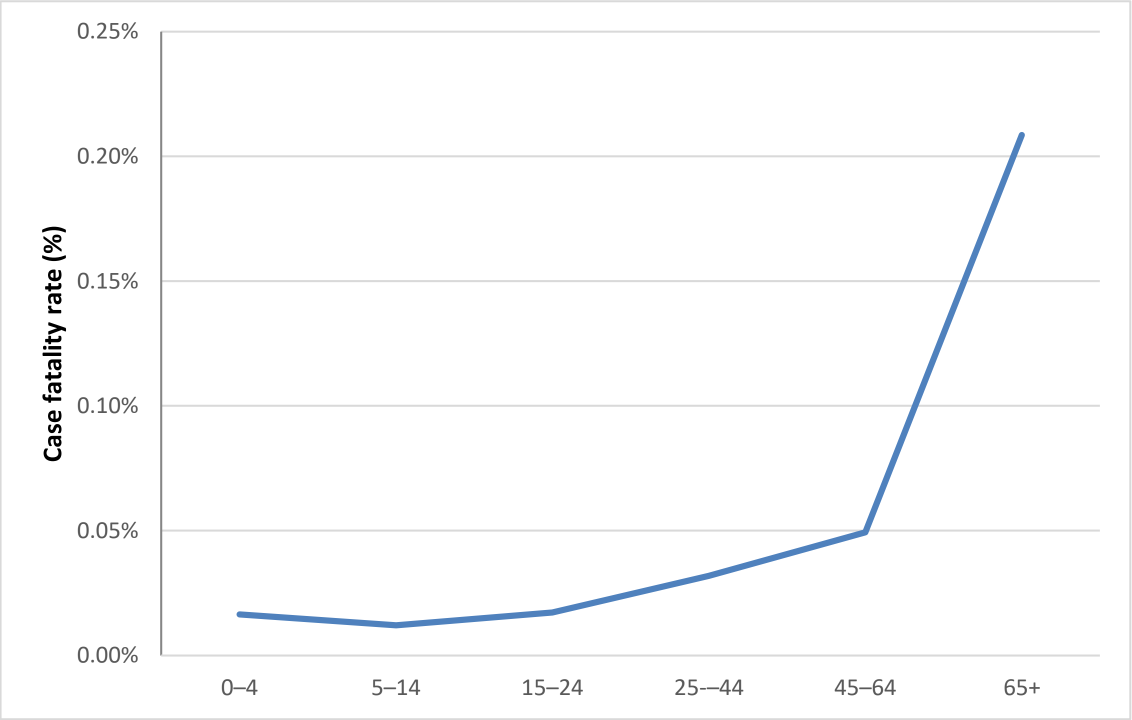

Source: Authors' own elaboration based on data from the SNVS and SNVS 2.0. National Ministry of Health.

Figure S4. Identified dengue virus serotypes by season. Argentina. Seasons 2010/11 to 2019/20. *N*= 9567.

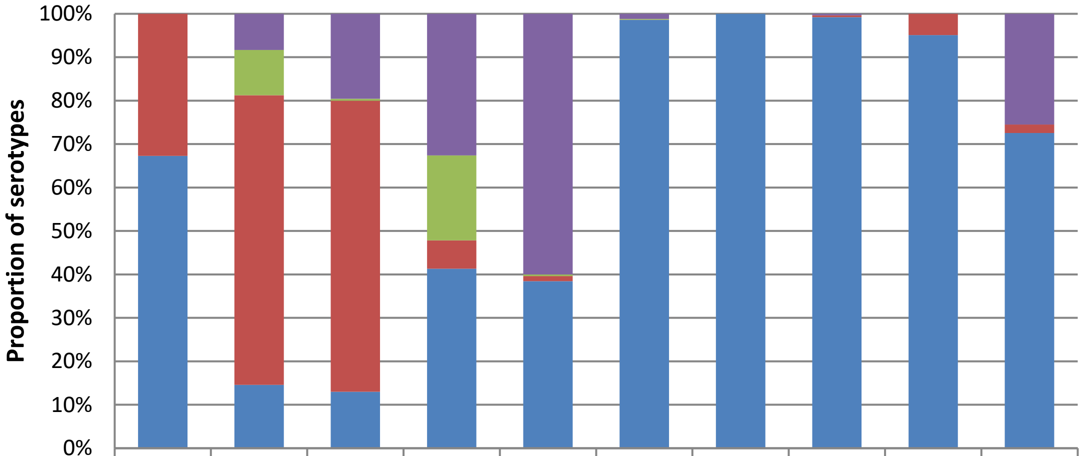

	2010/11	2011/12	2012/13	2013/14	2014/15	2015/16	2016/17	2017/18	2018/19	2019/20
DEN-4		4	92	15	153	39		1		1245
DEN-3		5	2	9	1	6				
DEN-2	35	32	315	3	3	1		1	3	94
DEN-1	72	7	61	19	98	3212	187	253	58	3541

Source: Authors' own elaboration based on data from the SNVS and SNVS 2.0. National Ministry of Health.
